# Association of body mass index with the risk of rheumatoid arthritis: a systematic review and meta-analysis

**DOI:** 10.3389/fmed.2025.1750640

**Published:** 2026-02-17

**Authors:** Fei Cao, Xiaohong Kang, Weizhuo Wang, Shengli Huang

**Affiliations:** 1 Department of Orhopedics, The Second Affliated Hospital of Xi’an Jiaotong University, Xi’an, China; 2 Department of Orhopedics, Pingdingshan First People’s Hospital, Pingdingshan, China; 3 Department of Oncology, The First Affiliated Hospital of Henan Medical University, Xinxiang, China

**Keywords:** body mass index, meta-analysis, rheumatoid arthritis, risk, systematic review

## Abstract

**Background:**

Rheumatoid arthritis (RA) is a chronic systemic autoimmune disease of unknown etiology. Numerous studies have investigated the association between body mass index (BMI) and RA risk, but findings have been inconsistent.

**Objective:**

This study aims to comprehensively evaluate the association between different BMI categories and RA risk using a meta-analytic approach.

**Methods:**

We systematically searched PubMed, Embase, Web of Science, and the Cochrane Library from inception until September 2025 for observational studies investigating the association between BMI and RA onset. A random-effects model was used to calculate pooled odds ratios (ORs) and 95% confidence intervals (CIs) for the association between different BMI categories and RA risk. The robustness of the findings was evaluated through sensitivity analyses, subgroup analyses, and assessment of publication bias.

**Results:**

This meta-analysis included 20 observational studies (8 cohort and 12 case-control studies) with a total sample size of 568,889. Our findings indicated no significant association between underweight and RA risk (OR: 0.84, 95% CI: 0.70–1.01, *p* = 0.058). In contrast, both overweight (OR: 1.13, 95% CI: 1.06–1.19, *p* < 0.001) and obesity (OR: 1.25, 95% CI: 1.14–1.36, *p* < 0.001) were significantly associated with an increased risk of RA, with the association being particularly pronounced in female participants.

**Conclusion:**

This study demonstrates that overweight and obesity are robustly associated with a significantly increased risk of developing RA, particularly among females. In contrast, the association between underweight and RA risk remains inconclusive and warrants further investigation.

**Systematic review registration:**

INPLASY (Registration Number: INPLASY2025110039).

## Introduction

Rheumatoid arthritis (RA) is a systemic autoimmune disease characterized primarily by chronic, symmetrical synovitis of multiple joints, affecting approximately 17.6 million people worldwide (1). Its pathogenesis is complex, involving interactions between genetic susceptibility and environmental factors (2). RA not only causes joint pain, swelling, deformity, and significantly reduced quality of life but also affects multiple systems such as the cardiovascular and respiratory systems, increasing disability rates, cardiovascular event incidence, and all-cause mortality. This imposes a substantial burden on affected families and healthcare systems (3). Despite considerable advances in treatment, the etiology of RA remains incompletely understood, making the identification of modifiable risk factors crucial for primary prevention.

Among various environmental factors, obesity—a global public health issue—has garnered increasing attention due to its association with chronic inflammation and immune dysregulation (4). Obesity is now recognized not merely as excess fat storage but as a chronic low-grade inflammatory state driven by the secretion of various pro-inflammatory cytokines from adipose tissue (5). This inflammatory milieu may disrupt immune tolerance and promote autoimmune responses, providing a biological plausibility for the link between obesity and RA (6). Body mass index (BMI), a simple and commonly used indicator of weight relative to height, is widely employed to define overweight and obesity.

Numerous epidemiological studies have investigated the relationship between BMI and RA risk, yet their findings have been inconsistent. Although previous systematic reviews have synthesized evidence in this field (7–10), significant gaps remain. First, the most recent comprehensive meta-analyses included literature up to 2019, leaving nearly a decade of subsequent research un-synthesized (7–10). The inclusion of newer, larger cohort studies is essential to confirm and refine previous estimates. Second, prior analyses have predominantly focused on overweight, obesity, or continuous BMI, with the association between underweight and RA risk remaining poorly characterized and underpowered in earlier work. Third, a detailed evaluation of how study quality and the degree of confounding adjustment influence these associations is needed. Therefore, this study aims to perform an updated systematic review and meta-analysis of existing observational studies to quantitatively synthesize evidence regarding the association between different BMI categories and the risk of developing RA.

## Methods

### Data sources, search strategy, and selection criteria

This systematic review and meta-analysis was conducted in accordance with the Preferred Reporting Items for Systematic Reviews and Meta-Analyses (PRISMA) guidelines (11). The study protocol was registered on INPLASY (Registration Number: INPLASY2025110039). We included published observational studies—cohort and case–control studies—that investigated the association between BMI and RA development. No restrictions were applied regarding language or publication status.

A comprehensive search of PubMed, Embase, the Cochrane Library, and Web of Science was performed from their inception until September 2025 to incorporate the most recent research. The search strategy combined subject headings and free-text terms using key concepts: (“body mass index” OR “BMI” OR “overweight” OR “obesity”) AND (“rheumatoid arthritis” OR “RA”). The detailed search strategy for each database is provided in Supplementary File 1. Reference lists of included studies were manually screened to identify additional relevant studies.

Two researchers independently performed literature selection through a two-step process: (1) initial screening of titles and abstracts to exclude irrelevant records, and (2) full-text assessment of potentially eligible articles. Disagreements were resolved through discussion or by consulting a third researcher. Studies were included if they met the following criteria: (1) the study population had baseline BMI clearly measured, with follow-up for RA incidence (cohort studies) or appropriate case–control matching (case–control studies); (2) the exposure was BMI, classified using WHO or regionally accepted cut-off points: underweight (<18.5 kg/m^2^), normal weight (18.5–24.9 kg/m^2^), overweight (25.0–29.9 kg/m^2^), and obesity (≥30.0 kg/m^2^); (3) the primary outcome was incident RA, diagnosed according to internationally accepted criteria (e.g., 1987 ACR, 2010 ACR/EULAR); (4) the study provided or allowed calculation of effect estimates with 95% CIs; (5) the study design was observational (cohort or case–control).

### Data collection and quality assessment

Two independent researchers used a pre-designed data extraction form to collect key information from included studies: first author, publication year, country/region, study design, sample size (number of RA cases/controls), age range, sex distribution, BMI classification criteria, follow-up duration (for cohort studies), original effect estimates with 95% CIs, and adjusted confounding factors. After independent extraction, results were cross-checked, and discrepancies were resolved as in the literature screening.

The included studies employed varying BMI categorization schemes. To ensure comparability for meta-analysis, all effect estimates were harmonized to the standard WHO categories: underweight (<18.5 kg/m^2^), normal weight (18.5–24.9 kg/m^2^), overweight (25.0–29.9 kg/m^2^), and obesity (≥30.0 kg/m^2^). The harmonization process followed a pre-defined, hierarchical strategy: (1) for studies that reported effect estimates for ranges that aligned precisely or almost entirely with WHO categories, the provided estimate was used directly; (2) for studies that provided more granular categories or continuous risk estimates per unit BMI increase, we used the reported category midpoints and sample sizes (or variance estimates) to re-calculate pooled odds ratios for the standard WHO categories where possible, applying methods described by Hamling et al. (12). This required the study to report either the distribution of cases and non-cases across the original categories or sufficient data to approximate it; and (3) for studies that used a single non-standard cut-point close to a WHO boundary, the reported ‘high BMI’ group (≥24.0 kg/m^2^) was considered to encompass both the ‘overweight’ and ‘obese’ categories according to the regional Asian-specific guidelines it followed. For the primary analysis using WHO categories, the effect estimate for this combined group was assigned to the ‘overweight’ category, as it represents the lower risk threshold. This assignment was tested in a sensitivity analysis.

The methodological quality of included studies was assessed using the Newcastle-Ottawa Scale (NOS) (13). The NOS rates studies across three domains: selection of study groups (up to 4 points), comparability of groups (up to 2 points), and ascertainment of outcome (cohort studies) or exposure (case–control studies) (up to 3 points), for a total score of 0–9. This assessment was performed independently by two researchers, with disagreements resolved through discussion or by consulting a third researcher.

### Statistical analysis

Using the normal-weight group (18.5–24.9 kg/m^2^) as the reference, we pooled effect sizes for the associations between underweight, overweight, and obese groups and RA risk. The odds ratio (OR) with 95% CI was used as the summary effect measure. All meta-analyses were performed using a random-effects model (14). Heterogeneity among studies was assessed using Cochran’s *Q* test and the *I*^2^ statistic, with significant heterogeneity indicated by *I*^2^ ≥ 50% or a *Q* test *p*-value ≤0.10 (15). Sensitivity analysis was conducted using the “leave-one-out” method to examine the influence of individual studies on the pooled estimate (16). Subgroup analyses were performed based on study type, geographic region, participant sex, level of adjustment for confounders, and study quality. High adjustment was defined as study who multivariable model adjusted for more than age, sex, and smoking status. These are considered the three core, well-established confounders for the BMI-RA association. Differences between subgroups were assessed using interaction tests, assuming normal distribution of the data (17). Publication bias was visually inspected using funnel plots and quantitatively assessed using Egger’s and Begg’s tests (18, 19). A *p*-value <0.05 suggested potential publication bias, which was adjusted for using the trim-and-fill method (20). All statistical tests were two-sided, and a pooled *p*-value <0.05 was considered statistically significant. Analyses were conducted using STATA version 18.0 (StataCorp LLC, College Station, Texas, USA).

## Results

### Literature search

**Figure 1:**
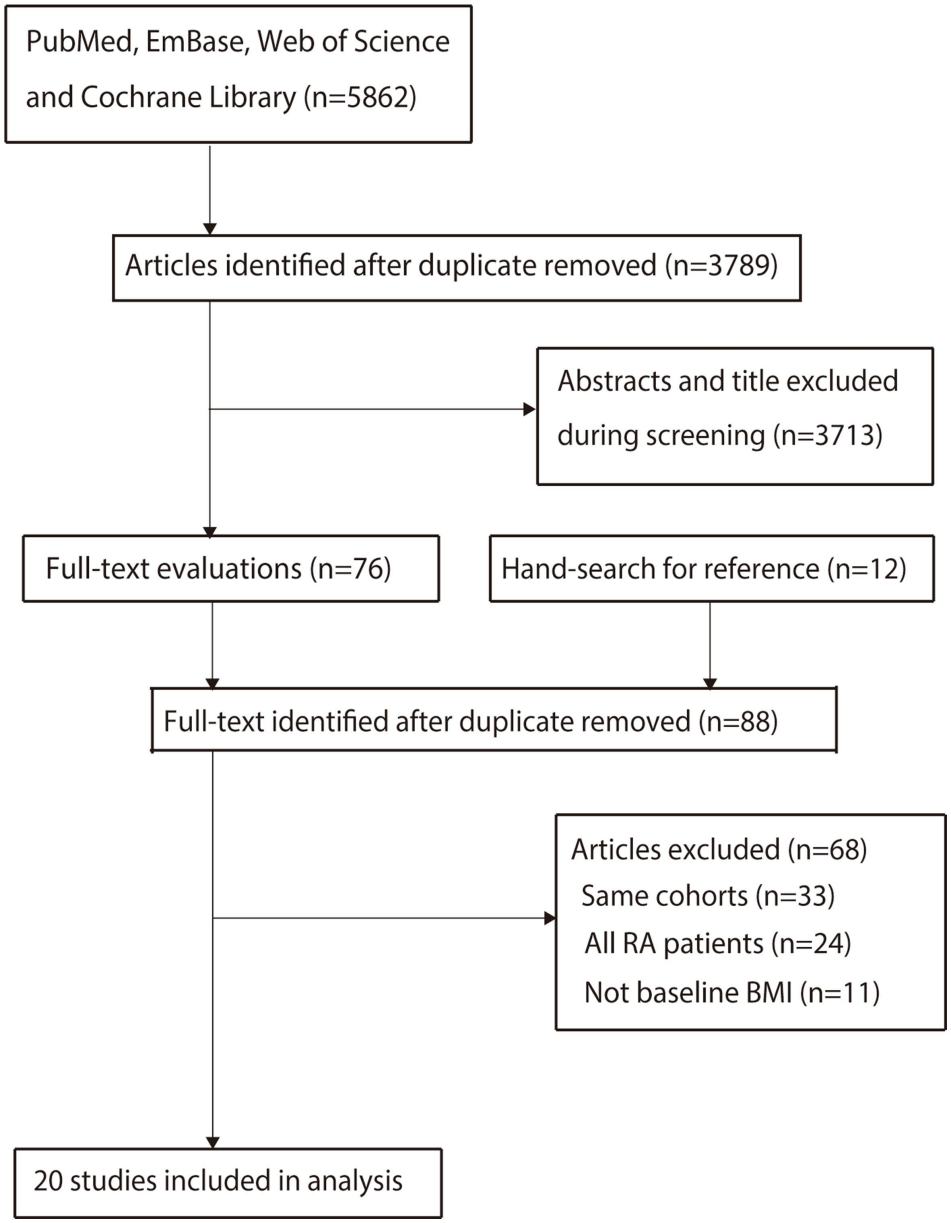
PRISMA flow diagram of the study selection process. This flowchart illustrates the process of identifying and selecting studies for the systematic review and meta-analysis. It details the number of records identified through database searching, the removal of duplicates, the screening of titles and abstracts, the retrieval and assessment of full-text articles, and the final inclusion of studies, with reasons for exclusions at each stage.

The electronic search yielded 5,862 records. After removing duplicates, 3,789 records underwent title/abstract screening, excluding 3,713 irrelevant studies. The remaining 76 articles underwent full-text assessment, leading to the exclusion of 56 articles due to overlapping cohorts (*n* = 24), study populations comprising only RA patients (*n* = 21), or exposure not being baseline BMI (*n* = 11). A manual search identified 12 additional records, but 9 reported on overlapping cohorts and 3 involved RA patients, leaving none for inclusion. Finally, 20 eligible observational studies (8 cohort and 12 case–control studies) were included in the analysis (21–40) (Figure 1).

### Characteristic of included studies

The 20 included studies, published between 1993 and 2025, consisted of 18 from Europe or America and 2 from Asia. Study and participant characteristics are summarized in Table 1. The total sample size was 568,889. Among cohort studies, there were 5,687 incident RA cases. In case–control studies, case groups ranged from 90 to 2,748 participants, and control groups from 93 to 5,725 participants. Quality assessment using the NOS indicated overall high methodological quality: 4 studies scored 9 points, 5 scored 8 points, 7 scored 7 points, and 4 scored 6 points.

**Table 1:** The baseline characteristics of identified studies and involved individuals.

Study	Study design	Country	Sample size	Age (years)	Sex	BMI category (kg/m^2^)	Adjusted factors	NOS
Heliovaara et al. (21)	Cohort	Finland	161/28364	30.0–69.0	Male	<25.0; 25.0–30.0; ≥ 30.0	Smoking, geographical region, type of population, marital status, social class, perceived, health, and age	9
Voigt et al. (22)	CC	USA	349/1457	18.0–64.0	Female	12.94–20.43; 20.44–22.51; 22.52–25.82; 25.83–52.86	Age, smoking	7
Symmons et al. (23)	CC	UK	90/93	18.0–70.0	Both	20.0–24.9; 25.0–29.9; 30.0–40.0	Smoking, social class	7
Uhlig et al. (24)	CC	Norway	347/5725	20.0–79.0	Both	<25.0; 25.0–29.9; ≥30.0	Age, sex, marital status, employment category, formal education, smoking	7
Cerhan et al. (25)	Cohort	USA	158/31336	55.0–69.0	Female	<23.4; 23.4–25.8; 25.9–29.2; >29.2	Age	7
Pedersen et al. (26)	CC	Denmark	505/752	18.0–65.0	Both	<18.5; 18.5–25.0; 25.0–30.0; >30.0	Birth year, year of RA diagnosis, gender	6
Rodriguez et al. (27)	CC	UK	559/4234	20.0–79.0	Both	<20.0; 20.0–24.9; 25.0–30.0; >30.0	Age, sex, calendar year, number of referrals, and visits to a primary care physician	8
Wesley et al. (28)	CC	Sweden	2748/3444	18.0–70.0	Both	<25.0; 25.0–30.0; ≥30.0	Sex, age, area of residence, smoking, alcohol, education	7
Crowson et al. (29)	CC	USA	813/813	≥18.0	Both	<30.0; ≥30.0	Age, sex, calendar year, smoking	6
Harpsoe et al. (30)	Cohort	Denmark	2430/75008	27.4–33.3	Female	<18.5; 18.5–25.0; 25.0–30.0; >30.0	Smoking, alcohol, parity, socio-occupational status	8
Lahiri et al. (31)	Cohort	UK	138/25455	40.0–79.0	Both	<25.0; 25.0–30.0; ≥30.0	Age, gender, smoking, breastfeeding, alcohol	9
Lu et al. (32)	Cohort	USA	826/109896; 355/108727	30.0–55.0; 25.0–42.0	Female	18.5–24.9; 25.0–29.9; ≥30.0	Age, community median income, smoking, alcohol, PA, parity, breastfeeding status, postmenopausal use, postmenopausal Hormone use	9
Ljung and Rantapää-Dahlqvist (33)	CC	Sweden	557/1671	25.0–74.0	Both	<25.0; 25.0–30.0; ≥30.0	Smoking, educational level, age, sex, year of health examination, cohort	7
Turesson et al. (34)	CC	Sweden	172/688; 290/1160	58.0	Both	18.5–25.0; 25.0–30.0; >30.0	Smoking, level of formal education, alcohol, socio-economic status	8
Fu et al. (35)	CC	China	400/400	18.0–75.0	Both	<23.9; >24.0	Sex	6
Wei et al. (36)	CC	China	403/128	30.0–75.0	Both	<25.0; 25.0–30.0; ≥30.0	Age, sex, waist circumference, erosive osteoarthritis, duration of disease, smoking	7
Linauskas et al. (37)	Cohort	Denmark	666/55037	50.0–64.0	Both	<18.5; 18.5–24.9; 25.0–29.9; ≥30.0	Age, smoking, socio-economic status, alcohol, PA and total intake of n-3 fatty acids	8
Kronzer et al. (38)	CC	USA	212/636	64.0	Both	<25.0; 25.0–30.0; ≥30.0	Crude	6
Salliot et al. (39)	Cohort	France	698/78452	40.0–65.0	Female	<18.5; 18.5–25.0; 25.0–30.0; >30.0	Ages at menarche, at menopause, number of full-term pregnancies, and baseline PA	8
Dong et al. (40)	Cohort	UK	255/27968	35.0–69.0	Female	18.5–25.0; 25.0–30.0; >30.0	Age, socio-economic status, marital status, menopausal status, hormone replacement therapy, and prevalence of cardiovascular diseases, cancer, or diabetes at recruitment, PA, smoking, alcohol, total energy intake, and Alternate Healthy Eating Index-2010	9

BMI, body mass index; CC, case–control; PA, physical activity; RA, rheumatoid arthritis.

### Underweight and RA risk

**Figure 2:**
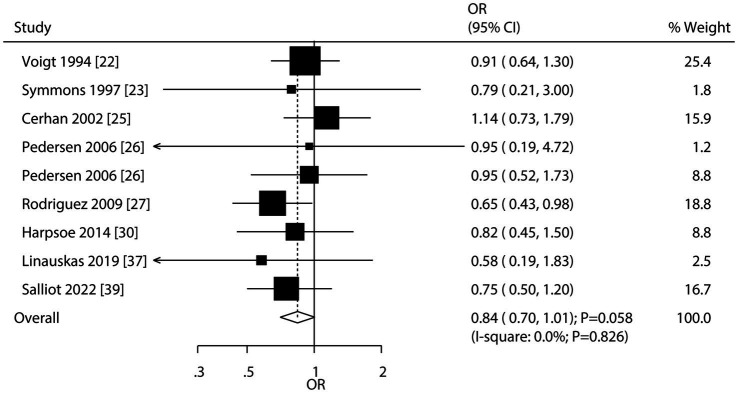
Forest plot for the association between underweight and risk of rheumatoid arthritis. The size of the data markers represents the weight of each study in the meta-analysis. The diamond at the bottom represents the pooled OR and 95% CI.

Nine studies reported on the association between underweight and RA risk. The meta-analysis found no significant association (OR: 0.84; 95% CI: 0.70–1.01; *p* = 0.058; Figure 2), with no evidence of heterogeneity (*I*^2^ = 0.0%; *p* = 0.826). However, sensitivity analysis suggested a potential significant association between underweight and a reduced RA risk Supplementary File 2. Subgroup analyses indicated that underweight was significantly associated with a lower RA risk in studies reporting highly adjusted effect estimates and in the subgroup of high-quality studies (Table 2). However, it is crucial to note that the subgroup analysis for underweight in males was based on only a single study, which severely limits statistical power and precludes any reliable sex-specific conclusion for this BMI category. Therefore, the observed point estimate for males in Table 2 should be interpreted with extreme caution. No significant publication bias was detected (Egger’s test *p* = 0.794; Begg’s test *p* = 0.917; Supplementary File 3.

**Table 2:** Subgroup analyses for the association of underweight, overweight, and obesity with the risk of rheumatoid arthritis.

BMI category	Factors	Subgroup	No of studies	OR and 95%CI	*p* value	*I^2^* (%)	*Q* statistic	Interaction test
Underweight	Country	Europe or US	8	0.84 (0.70–1.01)	0.058	0.0	0.826	–
		China	0	–	–	–	–	
	Study design	Case–control	4	0.82 (0.64–1.04)	0.094	0.0	0.769	0.698
		Cohort	4	0.88 (0.67–1.15)	0.335	0.0	0.500	
	Sex	Male	1	0.95 (0.19–4.73)	0.950	–	–	0.402
		Female	6	0.89 (0.73–1.10)	0.282	0.0	0.786	
	Adjusted level	High	4	0.71 (0.55–0.92)	0.010	0.0	0.901	0.081
		Crude or low	4	0.98 (0.76–1.52)	0.850	0.0	0.949	
	NOS	8/9	4	0.71 (0.55–0.92)	0.010	0.0	0.901	0.081
		6/7	4	0.98 (0.76–1.52)	0.850	0.0	0.949	
Overweight	Country	Europe or US	17	1.12 (1.06–1.19)	<0.001	0.0	0.563	0.810
		China	2	1.12 (0.81–1.56)	0.486	32.4	0.224	
	Study design	Case–control	11	1.09 (1.00–1.19)	0.047	0.0	0.757	0.323
		Cohort	8	1.14 (1.04–1.25)	0.006	18.6	0.277	
	Sex	Male	6	0.92 (0.79–1.08)	0.306	5.9	0.379	0.018
		Female	11	1.17 (1.09–1.26)	<0.001	0.0	0.822	
	Adjusted level	High	12	1.12 (1.04–1.20)	0.002	12.4	0.309	0.984
		Crude or low	7	1.13 (0.97–1.31)	0.111	0.0	0.855	
	NOS	8/9	9	1.12 (1.01–1.24)	0.029	32.6	0.138	0.401
		6/7	10	1.09 (1.00–1.20)	0.054	0.0	0.946	
Obesity	Country	Europe or US	18	1.25 (1.14–1.37)	<0.001	32.2	0.070	0.527
		China	1	1.02 (0.55–1.89)	0.950	-	-	
	Study design	Case–control	11	1.23 (1.08–1.41)	0.002	34.4	0.093	0.452
		Cohort	8	1.27 (1.12–1.45)	<0.001	27.0	0.204	
	Sex	Male	6	0.95 (0.65–1.38)	0.792	57.8	0.037	0.090
		Female	11	1.28 (1.18–1.39)	<0.001	0.0	0.667	
	Adjusted level	High	13	1.21 (1.08–1.35)	0.001	35.3	0.075	0.292
		Crude or low	6	1.35 (1.14–1.60)	<0.001	14.9	0.316	
	NOS	8/9	9	1.21 (1.02–1.39)	0.010	40.5	0.079	0.952
		6/7	10	1.27 (1.12–1.44)	<0.001	25.3	0.188	

### Overweight and RA risk

**Figure 3:**
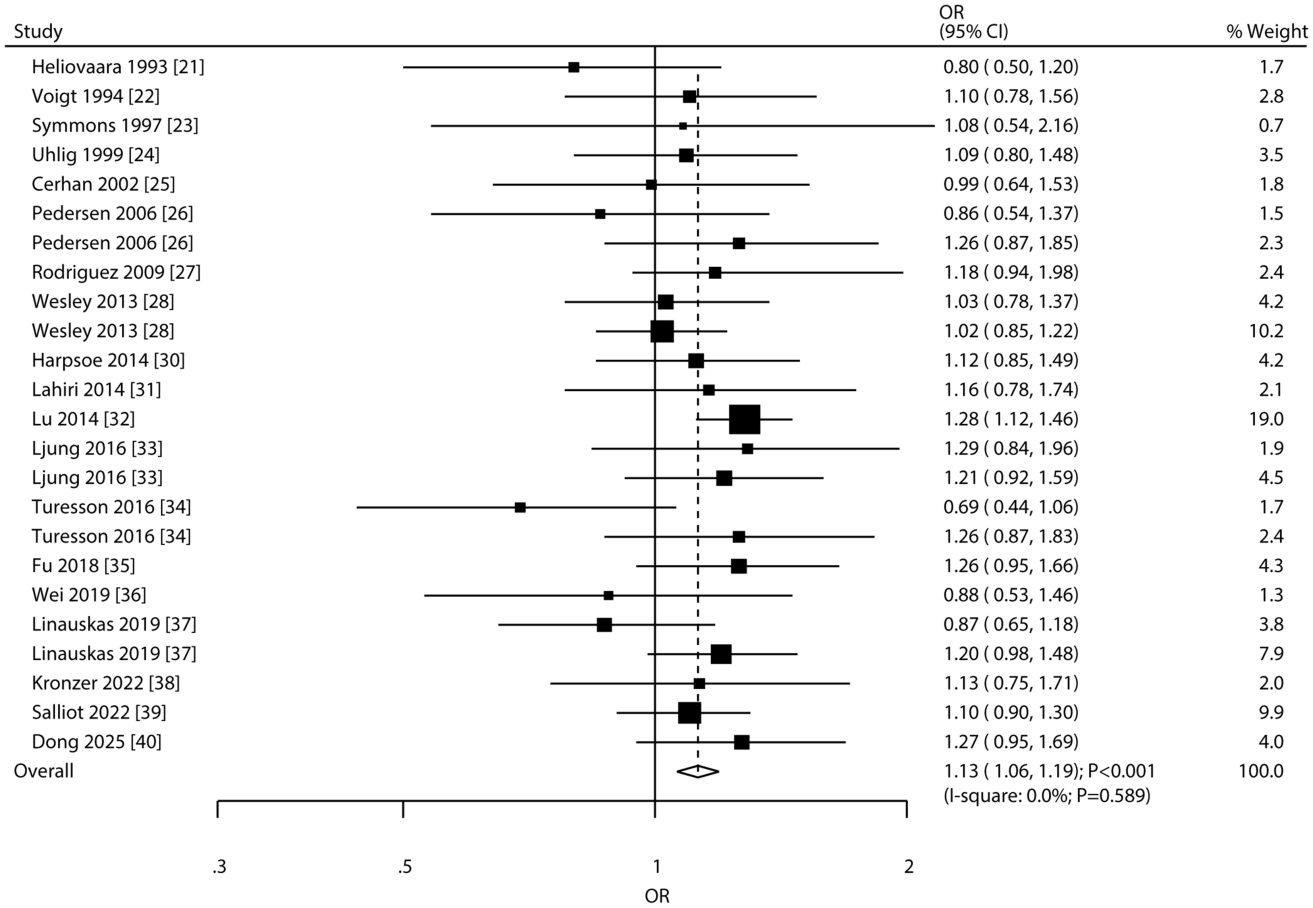
Forest plot for the association between overweight and risk of rheumatoid arthritis. The size of the data markers represents the weight of each study in the meta-analysis. The diamond at the bottom represents the pooled OR and 95% CI.

Nineteen studies reported on the association between overweight and RA risk. The meta-analysis revealed a significant association with increased RA risk (OR: 1.13; 95% CI: 1.06–1.19; *p* < 0.001; Figure 3), with no evidence of heterogeneity (*I*^2^ = 0.0%; *p* = 0.589). Sensitivity analysis confirmed the robustness of this association (Supplementary File 2). Subgroup analyses indicated a consistent positive association in most subgroups. However, no significant association was observed in studies conducted in Asia, those involving only male participants, analyses using crude or low adjustment levels, and studies with an NOS score of 6 or 7 (Table 2). Assessment for publication bias suggested potential significant bias (Egger’s test *p* = 0.038; Begg’s test *p* = 0.087; Supplementary File 3). The conclusion remained robust after adjusting for publication bias using the trim-and-fill method (20).

### Obesity and RA risk

**Figure 4:**
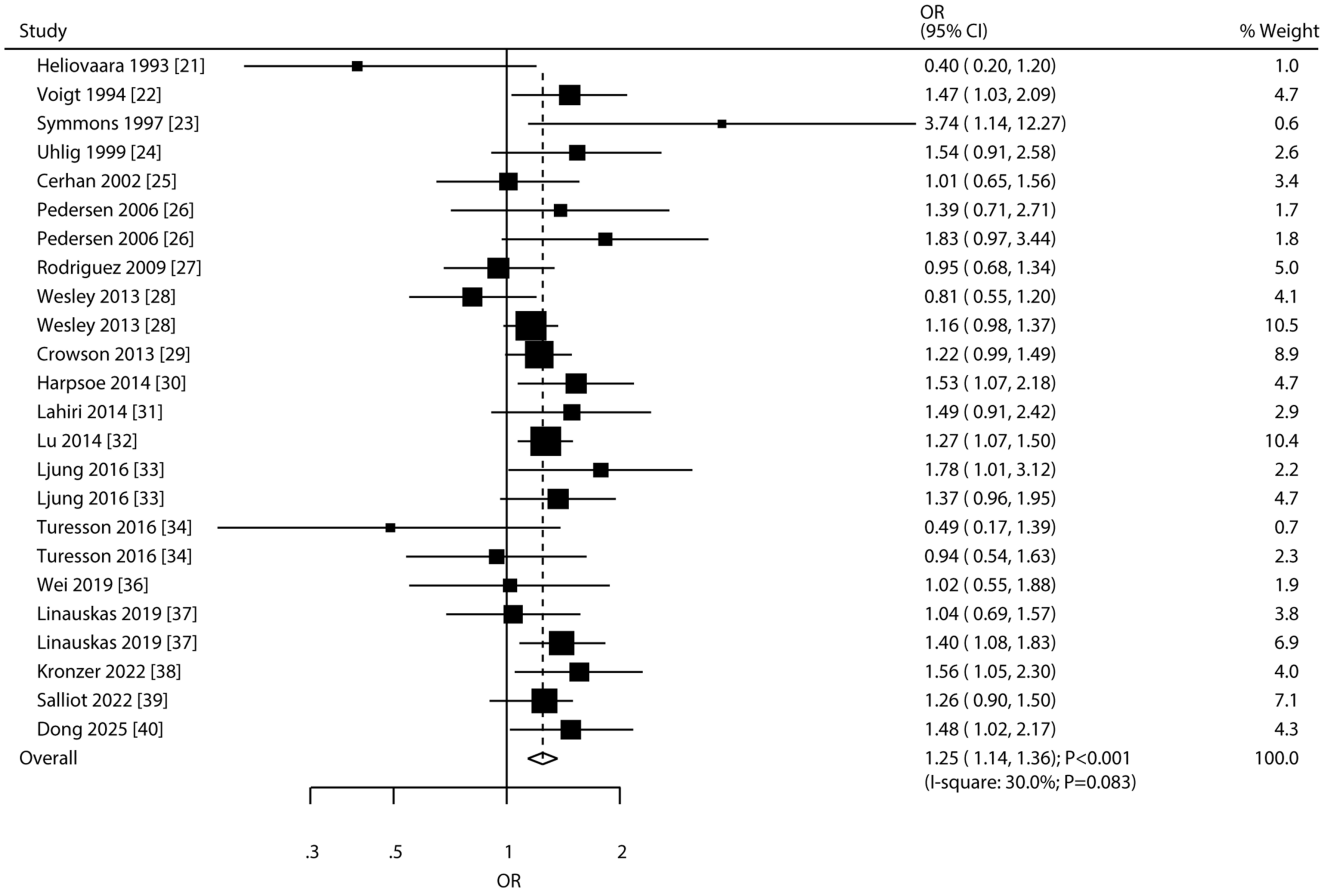
Forest plot for the association between obesity and risk of rheumatoid arthritis. The size of the data markers represents the weight of each study in the meta-analysis. The diamond at the bottom represents the pooled OR and 95% CI.

Nineteen studies reported on the association between obesity and RA risk. The meta-analysis revealed a significant association with increased RA risk (OR: 1.25; 95% CI: 1.14–1.36; *p* < 0.001; Figure 4), with moderate heterogeneity among studies (*I*^2^ = 30.0%; *p* = 0.083). Sensitivity analysis confirmed the robustness of this association (Supplementary File 2). Subgroup analyses demonstrated a consistent positive association in most subgroups. However, no significant association was observed in studies conducted in China or those involving exclusively male participants (Table 2). No significant publication bias was detected (Egger’s test *p* = 0.930; Begg’s test *p* = 0.710; Supplementary File 3).

## Discussion

This systematic review and meta-analysis, incorporating 20 observational studies (8 cohort and 12 case–control) published up to 2025 with a total sample size of 568,889, extends and refines the existing body of evidence on BMI and RA risk (7–10). Our findings confirm and strengthen the positive association between higher BMI (overweight and obesity) and increased RA risk, previously reported (7–9), by demonstrating its robustness in the most contemporary dataset and across high-quality studies. Notably, our analysis provides novel insights into the underweight category, an area less explored in prior syntheses. While Feng et al. (8, 9) and Ohno et al. (10) primarily reported on overweight/obesity or continuous BMI, we found that underweight was not significantly associated with RA risk overall, but showed a trend towards a protective effect in methodologically rigorous subgroups, a finding that warrants future investigation. Furthermore, our stratified analyses by study quality (NOS score) and adjustment level offer a more nuanced understanding of how methodological heterogeneity may influence the observed associations, particularly for underweight.

This study confirms the promotive effect of overweight and obesity on RA onset, aligning with the biological mechanism of “obesity as a chronic low-grade inflammatory state” (4, 5). Adipose tissue, particularly visceral fat, secretes substantial pro-inflammatory cytokines and adipokines. These mediators can break immune tolerance, activate autoreactive T and B cells, and directly promote synovial hyperplasia and inflammatory infiltration—key processes in RA pathogenesis (41). The higher pooled OR for obesity compared to overweight, while not establishing a formal dose–response relationship, suggests a potential graded association between increasing BMI and RA risk. This trend is consistent with previous studies reporting that each 5 kg/m^2^ increase in BMI is associated with an approximately 3–13% increase in RA risk (7, 8), further supporting the hypothesis that obesity-related inflammatory burden accumulates with rising BMI.

Our analysis provides a novel contribution by specifically evaluating the underweight category, which has been less clearly addressed in earlier meta-analyses (7–10). Overall, underweight was not significantly associated with RA risk. However, the significantly reduced risk observed in subgroups of studies with highly adjusted effect estimates and higher methodological quality is intriguing. This pattern may be explained by two factors. First, from a biological perspective, individuals with low body weight have reduced adipose tissue, potentially leading to lower pro-inflammatory cytokine secretion and a decreased probability of initiating autoimmune responses. Second, the potential for reverse causation bias must be considered. Undiagnosed early RA symptoms could lead to reduced physical activity and appetite loss, resulting in weight loss before formal diagnosis. Although our analysis used baseline BMI data, studies with shorter follow-up periods might still be susceptible to this bias. Furthermore, this nuanced finding highlights the importance of study quality in assessing this particular association and underscores why a dedicated analysis was needed beyond the scope of prior reviews. Finally, the finding regarding underweight is based on only 9 studies, and the limited sample size necessitates caution in interpretation. This caution is especially warranted for sex-specific interpretations. As noted in the results, the subgroup estimate for underweight in males was derived from a single study, providing insufficient evidence to characterize this association or to compare it reliably with the estimate for females. Therefore, while the overall null association for underweight appears robust, definitive conclusions regarding potential sex differences within the underweight category cannot be drawn from the current available evidence and require future studies with adequate sample sizes in both sexes.

This systematic review and meta-analysis, incorporating the most up-to-date evidence, strengthens the conclusion that overweight and obesity are modifiable risk factors for RA, particularly among females. The heightened risk observed in females may be explained by several mechanisms. During reproductive years, higher estrogen levels, while generally anti-inflammatory, can be paradoxically pro-inflammatory in the context of obesity. Adipose tissue secretes aromatase, which converts androgens to estrogen, leading to abnormally high levels that can activate the NF-κB pathway and exacerbate synovial inflammation (42). Conversely, in postmenopausal women, the sharp decline in endogenous estrogen is accompanied by a compensatory increase in pro-inflammatory cytokine secretion from adipose tissue, further amplifying the obesity-associated risk (43). In men, testosterone may confer a protective effect by activating the androgen receptor to suppress production of pro-inflammatory molecules like IL-6 and leptin while promoting anti-inflammatory factors such as IL-10 (44). This may partially counteract the inflammatory burden of obesity, potentially explaining the attenuated association between BMI and RA risk observed in the male subgroup. The regional differences, primarily the non-significant association found in the two studies conducted in China, should be interpreted with caution due to limited data from non-European/American populations and require further validation. Finally, the influence of adjustment levels on the observed associations suggests that potential interactions between BMI and confounding factors, such as smoking and alcohol consumption, warrant further investigation (45, 46).

This study has several limitations. First, as it incorporates both case–control and cohort studies, the findings are susceptible to recall and selection biases inherent in these designs. Second, the observational nature cannot eliminate residual confounding or definitively establish causality between obesity and RA. Third, BMI reflects only weight relative to height and cannot differentiate fat distribution from muscle mass, whereas abdominal obesity may have stronger pro-inflammatory effects (47). Fourth, with only two studies from Asia, it is difficult to accurately represent the true association within Asian populations. Fifth, although we employed a systematic strategy to harmonize diverse BMI categorizations across studies to the WHO standard, some degree of misclassification is inevitable. Studies using regional cut-offs or non-standard categories introduce heterogeneity in the exposure definition. Finally, as the analysis is based on published studies, the inability to access unpublished data may introduce publication bias.

## Conclusion

This study confirms that overweight and obesity are significantly associated with an increased risk of RA, with a notable sex-specific disparity: the risk elevation was more pronounced in females, while no significant association was observed in males. Underweight showed no significant overall association with RA risk. These findings provide robust evidence for the primary prevention of RA and underscore the importance of developing sex-specific weight management strategies, laying a foundation for future research into targeted preventive interventions.

## Data Availability

The original contributions presented in the study are included in the article/supplementary material, further inquiries can be directed to the corresponding author.
