# Supplementary material for: Association of body mass index with the risk of rheumatoid arthritis: a systematic review and meta-analysis
Source: Front Med (Lausanne). 2026 Feb 17;12:1750640. doi: 10.3389/fmed.2025.1750640 (PMC12953552; doi:10.3389/fmed.2025.1750640)
Supplement: Supplementary file 1 [file Data_Sheet_1.docx]

**PubMed:**  (“Obesity”[Mesh] or “Obesity” or “Obese” or “Body Mass Index”[Mesh] or “Body Mass Index” or “Overweight”[Mesh] or “Overweight”) and (“Arthritis, Rheumatoid”[Mesh] or “Rheumatoid Arthritis”).

**EmBase:** (exp “Rheumatoid Arthritis” or “Rheumatoid Arthritis”) and (exp “Obesity” or “Obesity” or exp “Body Mass Index” or “Body Mass Index” or Exp “Overweight” or “Overweight”).

**Cochrane library:** ("Obesity"[Mesh] OR "Obesity" OR "Obese" OR "Body Mass Index"[Mesh] OR "Body Mass Index" OR "BMI" OR "Overweight"[Mesh] OR "Overweight") AND ("Arthritis, Rheumatoid"[Mesh] OR "Rheumatoid Arthritis" OR "RA")

**Web of Science:** TS=("Rheumatoid Arthrit*" OR RA) AND (Obes* OR "Body Mass Index" OR BMI OR Overweight).
