# Supplementary material for: Association of body mass index with the risk of rheumatoid arthritis: a systematic review and meta-analysis
Source: Front Med (Lausanne). 2026 Feb 17;12:1750640. doi: 10.3389/fmed.2025.1750640 (PMC12953552; doi:10.3389/fmed.2025.1750640)
Supplement: Supplementary file 3 [file Data_Sheet_3.docx]

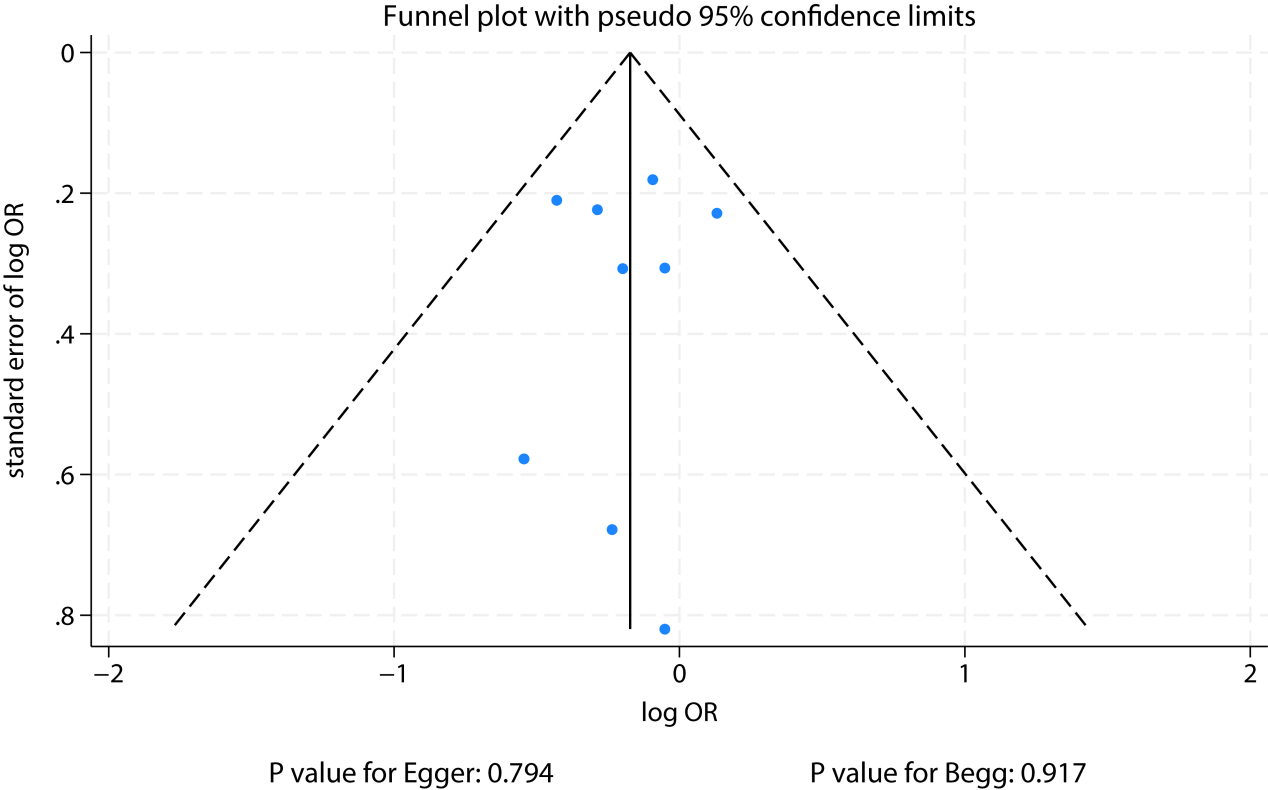


Figure S1. Funnel plot for the association of underweight with the risk of RA


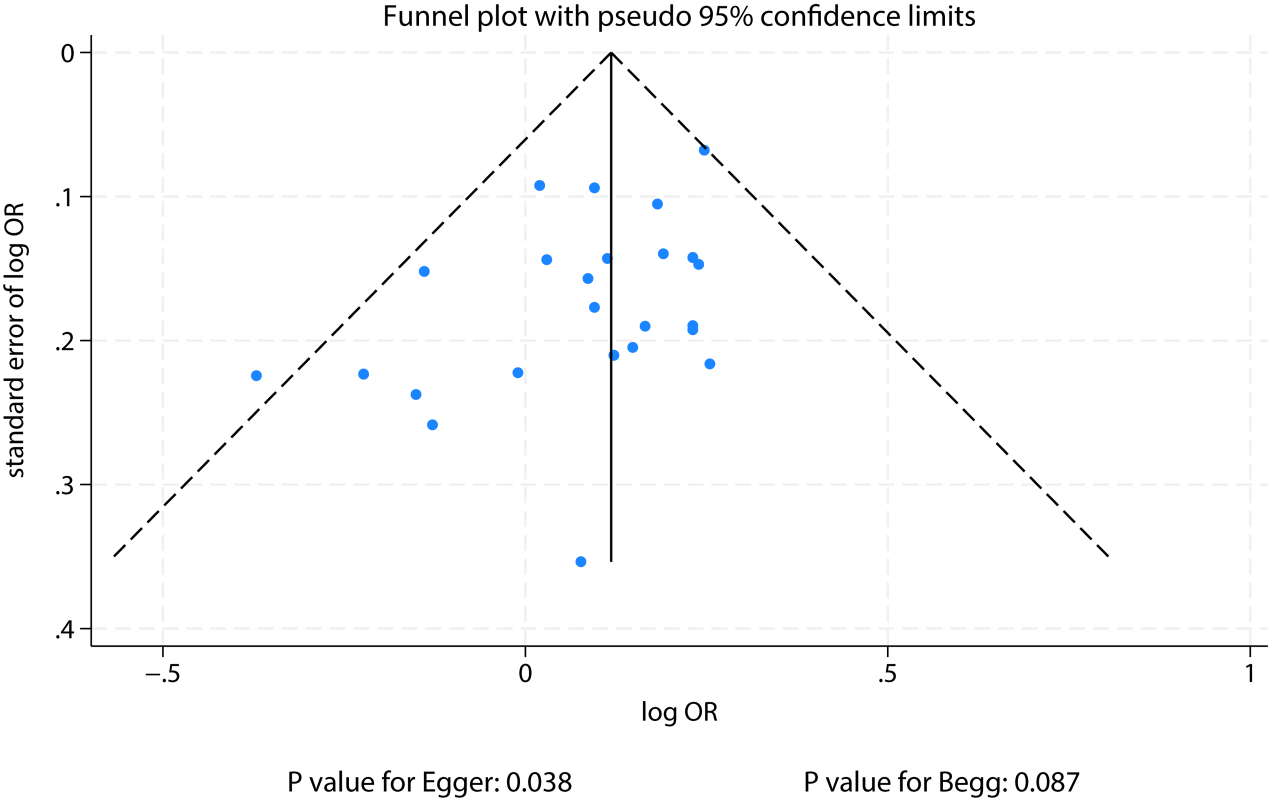


Figure S2. Funnel plot for the association of overweight with the risk of RA


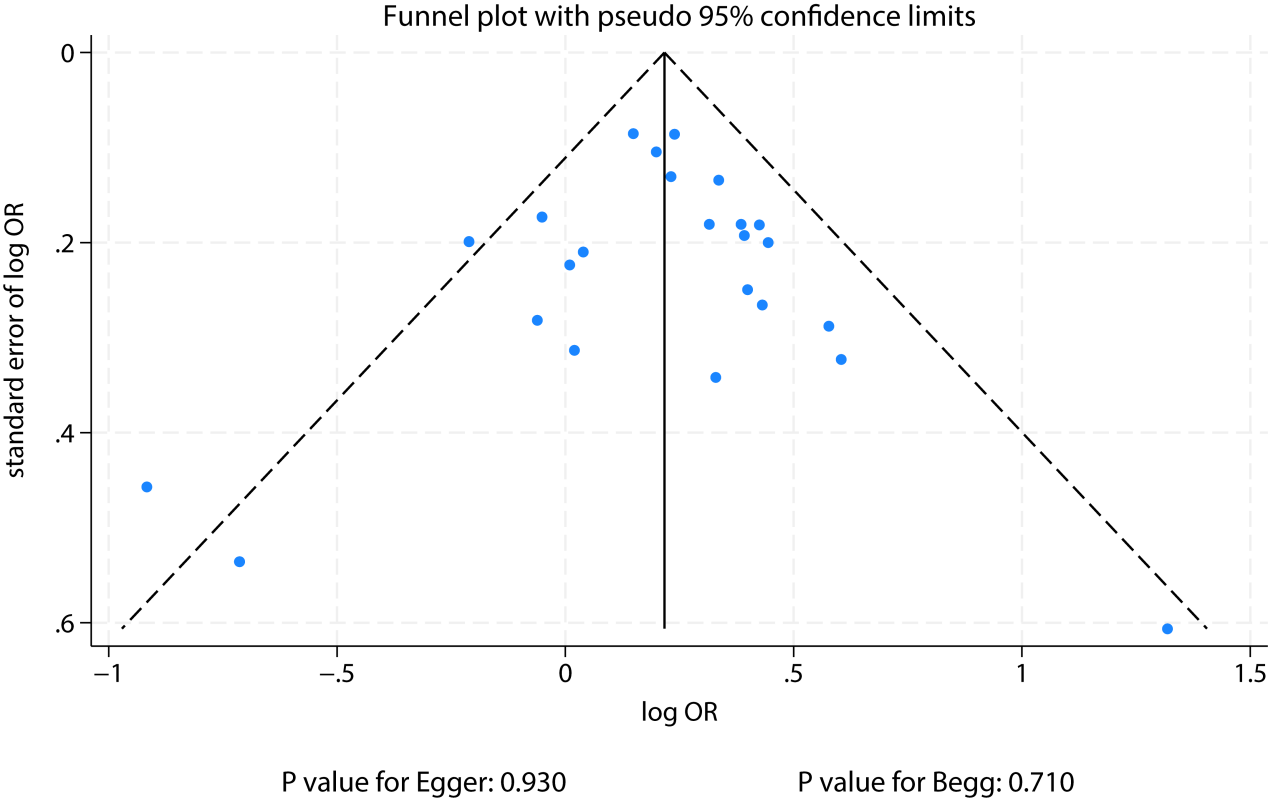


Figure S3. Funnel plot for the association of obesity with the risk of RA
